# Supplementary material for: NMDA receptor activation induces long-term potentiation of glycine synapses
Source: PLoS One. 2019 Sep 9;14(9):e0222066. doi: 10.1371/journal.pone.0222066 (PMC6733442; doi:10.1371/journal.pone.0222066)
Supplement: Attachment — Submitted filename: review comments_PONE19003.docx [file pone.0222066.s001.docx]

Recommendation: Minor revision

Comments to Author: Ref. PONE-D-19-19003

Title: NMDA receptor activation induces long-term potentiation of glycine synapses

Overview and general recommendation:

This manuscript mechanistically revealed how glycine synapses is potentiated by activation of NMDAR. I found that the paper is clearly written in general and much of it is well described. I feel confident that the authors performed careful and thorough experiments to test their hypothesis. However, the current version needs to be revised for publication due to the following reasons:

1. Missing certain literature citations in discussion part.

2. Please rewrite and emphasis the significance of this study in the manuscript.
